# MTL-P300 as a marker of the epileptogenic zone and hippocampal functionality in the presurgical evaluation of temporal lobe epilepsy: a systematic review

**DOI:** 10.1055/s-0042-1758643

**Published:** 2022-12-29

**Authors:** Daniela de Andrade Morange, Martha Silvia Martinez-Silveira, Magali Teresópolis Reis Amaral, Agnès Trebuchon

**Affiliations:** 1 Université d'Aix-Marseille, Institut de Neurosciences des Systèmes, Marseille, France.; 2 Hospital Universitário Prof. Edgard Santos, Serviço de Neurofisiologia Clínica, Salvador BA, Brazil.; 3 Fundação Oswaldo Cruz (Fiocruz), Instituto Gonçalo Muniz, Salvador BA, Brazil.; 4 Universidade Estadual de Feira de Santana, Salvador BA, Brazil.; 5 Assistance Publique - Hôpitaux de Marseille, Hôpital de la Timone, Service de Neurophysiologie Clinique, Marseille, France.

**Keywords:** Evoked Potentials, Event-Related Potentials, P300, Epilepsy, Temporal Lobe, Hippocampus, Potenciais Evocados, Potencial Evocado P300, Epilepsia do Lobo Temporal, Hipocampo

## Abstract

**Background** In the past twenty years, there has been an increasing interest among neuroscientists and physicians in mapping the cortical areas involved in the epileptogenic zone (EZ) through event-related potentials (ERPs) that enable the evaluation of the functional preservation of these areas. The present review is an update on publications on this topic.

**Objective** To investigate the accuracy of the cognitive evoked of the medial temporal lobe P300 (MTL-P300) potential in detecting the EZ in temporal lobe epilepsy (TLE).

**Methods** The systematic review of articles on the PubMed, Embase and Lilacs databases was conducted between February and December 2020. Articles published in English from 1985 to December 2020 were included. Additional studies were identified by searching the references of the selected studies and review articles. The studies were included for the following reasons: in-depth intracranial electroencephalography (iEEG) analysis of hippocampal activity; investigations of patients with TLE; and correlations between regarding the ERP results obtained in the temporal regions (MTL-P300) and the EZ.

**Results** In the three studies analyzed, the authors were able to define the laterality of the EZ during the preoperative investigation through the MTL-P300 results. The sensitivity of this method was of ∼ 70% to 80%, and the specificity between 70% and 94.7%. One of the limitations of the present review was the low number of studies.

**Conclusion** There is evidence that the reduced amplitude of the MTL-P300 has high specificity in identifying the EZ, and this is a good marker for diagnosis in unilateral TLE. The low sensitivity and negative likelihood ratios negative that a normal MTL-P300 response does not exclude the epileptogenicity of the hippocampus.

## Resumo

**Antecedentes** Nos últimos 20 anos, tem havido um crescente interesse de neurocientistas e médicos em mapear áreas corticais envolvidas na zona epileptogênica (ZE) por meio de potenciais relacionados a eventos (PREs), que permitem avaliar a preservação funcional dessas áreas. Esta revisão é uma atualização das publicações sobre esse tema.

**Objetivo** Investigar a acurácia do potencial evocado cognitivo do lobo temporal medial P300 (medial temporal lobe P300, MTL-P300, em inglês) na detecção da ZE em casos de epilepsias do lobo temporal (ELT).

**Métodos** A revisão sistemática de artigos nas bases de dados PubMed, Embase e Lilacs foi realizada entre fevereiro e dezembro de 2020. Foram incluídos artigos publicados em inglês de 1985 a dezembro de 2020. Estudos adicionais foram identificados por meio de busca nas referências dos estudos selecionados e artigos de revisão. Os estudos foram incluídos pelas seguintes razões: análise detalhada por meio de eletroencefalografia intracraniana (iEEG) da atividade hipocampal; investigações de pacientes com ELT; e correlações entre os resultados de ERP obtidos nas regiões temporais (MTL-P300) e na ZE.

**Resultados** Nos três estudos analisados, os autores foram capazes de definir a lateralidade da ZE durante a investigação pré-operatória por meio dos resultados do MTL-P300. A sensibilidade deste método foi de 70% a 80%, e a especificidade, entre 70% e 94.7%. Uma das limitações desta revisão foi o baixo número de estudos.

**Conclusão** Há evidências de que a amplitude reduzida do MTL-P300 tem alta especificidade na identificação da ZE, e este é um bom marcador para o diagnóstico na ELT unilateral. A baixa sensibilidade e a razão de verossimilhança negativa indicam que a resposta MTL-P300 normal não exclui a epileptogenicidade do hipocampo.

## INTRODUCTION

Currently, the preoperative period in cases of temporal lobe epilepsy (TLE) is characterized by investigations of the interictal activity and recording of electroclinical seizures to define the epileptogenic zone. Studies are performed in association with these investigations to map aspects related to cortical functionality, among them the oddball paradigm, a target detection task that, when registered through intracranial electroencephalography (iEEG) by hippocampal electrodes, generates the event-related potential of the medial temporal lobe P300 (MTL-P300). In the present study, we have reviewed the articles that analyzed the MTL-P300 as a tool to identify the epileptogenic zone (EZ) through intracranial electrodes. The MTL-P300 is a cognitive potential obtained through different modalities of visual, auditory, and somatosensory stimuli during the performance of the oddball paradigm. It consists of a simple paradigm to detect infrequent events considered relevant among other frequent events, as well as other infrequent ones designated as irrelevant. During the standard oddball paradigm (two stimuli) and novelty oddball paradigm (three stimuli), patients must distinguish between two or three stimuli responding to the target stimulus (pressing a button and mentally counting the number of targets) and refraining from responding to the frequent or irrelevant stimuli. When the patient detects the target stimulus, a hippocampal response of greater amplitude appears in relation to the resulting response during the frequent irrelevant stimulus. This component of the hippocampus was called MTL-P300 to indicate its local generation in the medial temporal lobe (MTL). 1 2 3

It is well known that the MTL is involved in the formation of memory and that the hippocampus participates in the process of storing information. 4 The TLE surgery exposes the patient to the risk of memory complications. The preservation of hippocampal functionality is assessed through preoperative investigations using structural and functional methods. In this context, the study of the MTL-P300 potential can be proposed during iEEG explorations. Some studies have described the association between the MTL-P300 unilaterally absent on the side of the onset of seizures 5 6 and the presence of hippocampal sclerosis. 7 It is known that the presence of a response to a rare stimulus (MTL-P300) significantly greater than the response to a frequent stimulus is associated with normal hippocampal morphology. 7 However, in cases without hippocampal sclerosis, it is not clear whether this test has good accuracy in detecting the hippocampus involved in the EZ. Then, the oddball paradigm makes it possible to obtain the MTL-P300, enabling the bilateral functional mapping of the hippocampi, and consequently clarifying the risk of resection of a hippocampus with preserved functionality, especially in the dominant hemisphere, as well as the functional reserve ensured by the hippocampus that is contralateral to the EZ.

In this sense, a systematic review is relevant to better identify the evidence on the accuracy of this marker to define the EZ and, consequently, to evaluate the functionality of the hippocampus. In the present systematic review, the population of interest are patients with drug-resistant TLE, candidates for epilepsy surgery, investigated through iEEG with electrodes in the hippocampi and through the oddball paradigm to detect the MTL-P300. The analysis of amplitudes, latencies, and the frequency spectrum of the MTL-P300 on the side of the EZ in relation to the non-epileptogenic side will be taken into account, as well as results of verbal memory performance when correlated to the results of the MTL-P300. The main parameter of interest is the accuracy of MTL-P300 in detecting the EZ and, as a secondary result, to correlate this ERP to the verbal memory performance results, if available. Observational studies in humans were considered for inclusion: case series, cohort studes, and cross-sectional studies.

## METHODS

The Preferred Reporting Items for Systematic Review and Meta-analysis Protocols (PRISMA-P) statement 8 was used. The study protocol was registered on the International Prospective Register of Systematic Reviews (PROSPERO) website under number CRD42021235450.

The search and selection of studies were performed according to the Population, Intervention, Comparison, Outcome, and Study Type (PICOS) strategy:

(1) Population – the patients included had focal drug-resistant epilepsy;
(2) Intervention/Exposure – they were submitted to an iEEG to investigate the hippocampus in the preoperative stage, and underwent to the oddball paradigm;
(3) Comparison – hippocampal MTL-P300 of the epileptogenic focus versus the MTL-P300 response of the contralateral hippocampus;
(4) Outcome – studies that evaluate the MTL-P300 as an EZ marker;
(5) Study type – descriptive, cohort, and cross-sectional studies.

The question of the present systematic review was: what is the accuracy of the cognitive evoked potential MTL-P300 in detecting the EZ in TLE?

### Searching the literature

The literature search was performed in the PubMed, Embase and Lilacs databases in December 2020, and a complementary search on Google Scholar was also performed. A manual search was performed in the references of the selected articles to find other potentially eligible articles.

The keywords used were: *intracranial electroencephalography* ; *iEEG* ; *medial temporal lobe* ; *hippocampus* ; *oddball* ; *evoked potentials* ; *event-related potentials* ; *P300* ; *P3* ; *ERP* ; and *MTL-P300* . The search was performed systematically in the same way for all online databases adapting the strategy.

### MEDLINE/PubMed search strategy

1) *Electroencephalography* [MeSH Terms] OR *Brain Mapping* [MeSH Terms] OR *intracranial electroencephalography* OR *iEEG* OR *SEEG* OR *Stereo elctroencephalography* OR *Intracranial EEG* OR *Stereo-electroencephalography* OR *stereo-EEG* OR *Stereoelectroencephalography* ;
2) *Event-Related Potentials, P300* [MeSH Terms] OR Event-Related Potential* OR *P3* OR *P3b* OR *P300* OR *MTL-P300* OR *ERPs* OR *oddball* OR *detection paradigm* OR *event-related potential* ;
3) *Epilepsy* [MeSH Terms] OR epilepsy* [TW] OR *hippocampus* OR *temporal lobe epilepsy* OR *epileptic zone* ; and
4) 1 AND 2 AND 3.

### Eligibility criteria and selection of studies

Articles published in English from 1985 to December 2020 were included. Articles published before 1985 were not included because they use a different methodology than the current ones. Observational studies in humans were considered for inclusion: case series, cohort studies, and cross-sectional studies. The identified titles, abstracts, and full-text articles were read and blindly selected by two independent reviewers. In the event of disagreement, a third reviewer resolved the impasse.

We excluded studies with EEG recordings on the surface of the skull to concentrate the research only on results through the iEEG recordings. In the following steps, studies that did not correlate MTL-P300 to the EZ were excluded.

Regarding the articles found that were written by the same group of authors, those with a greater number of patients were included, to the detriment of those with a smaller number of patients, as long as the article provided the necessary baseline data to calculate the accuracy of the MTL-P300 in lateralizing the EZ. Therefore, articles that did not show abnormal MTL-P300 responses concordant with the lateralization of the EZ, according to in-depth recording analysis of the iEEG, not enabling the calculation of diagnosis accuracy, using sensitivity, specificity, and likelihood ratio tests, were also excluded from the analysis.

### Assessment of methodological quality

We applied the Joanna Briggs Institute (JBI) 9 scale to the cross-sectional studies to evaluate studies included in terms of methodological quality ( Table 1 ), since only this type of design was found in the searches. The scale consists of eight items. The analysis was performed by two reviewers, and disagreements were solved by a third examiner.

**Table 1:** Methodological classification assessed by the Joanna Briggs Institute scale for cross-sectional studies

Authors/year	Criteria*								
	1	2	3	4	5	6	7	8	Total
Puce et al., 1989 7	Y	Y	Y	Y	N	N	Y	Y	6
Meador et al., 1992 20	Y	U	Y	Y	N	N	Y	Y	5
Grunwald et al., 1995 19	Y	Y	Y	Y	NA	NA	Y	Y	6

Abbreviations: N, no; NA, not applicable; U, nuclear; Y, yes.

Note: *1: Criteria for inclusion in the sample defined; 2: subjects and the setting described; 3: exposure measured in a valid way; 4: standard criteria to measure the condition; 5: confounding factors identified; 6: strategies to deal with confounding factors stated; 7: outcomes measured in a valid way; 8: appropriate statistical analysis.

### Data collection and analysis

The data of the articles were extracted and classified using a data extraction spreadsheet (Microsoft Excel, Microsoft Corp., Redmond, WA, United States) created based on the relevant information at the beginning of the research and improved according to the inclusion of the studies. Two independent researchers read the full text of the selected studies and extracted the data in pairs.

The analysis of the results was based on the agreement between the abnormal response of the MTL-P300 in the oddball paradigm and the EZ defined through the clinical and electrographic diagnosis from the recordings with depth electrodes (iEEG), which is considered the gold standard method in patients with unilateral TLE. The sensitivity of the method to separately define the laterality of the EZ in TLE ( left or right), as well as in relation to the presence or absence of hippocampal sclerosis was investigated and discussed in the articles.

## RESULTS

**Figure 1:**
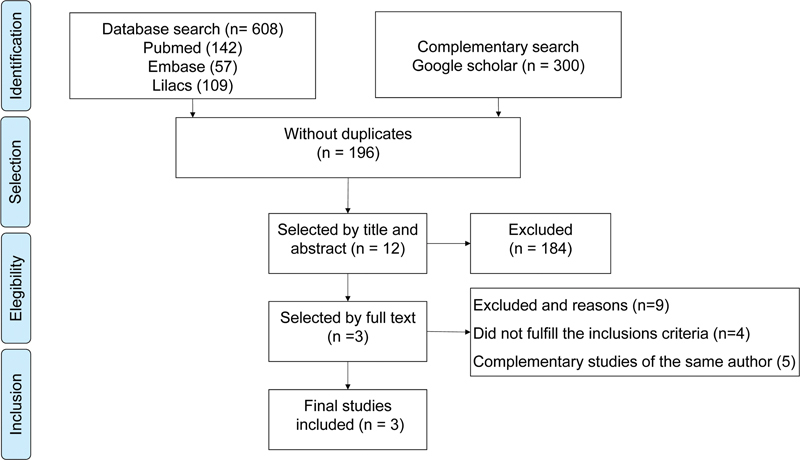
Flowchart of the search for and selection of studies.

From the total of 608 articles, the search resulted in 308 citations in the databases represented in the flowchart shown in Figure 1 . The complementary search on Google Scholar resulted in 300 articles, and no study was added based on the manual searches. After the exclusion of duplicate citations, 196 citations were submitted to a selection process based on the title and abstracts, and 12 citations remained. The full text of these 12 selected citations was examined in more detail. Nine studies were excluded because they were either complementary studies from the same group of authors, 10 11 12 13 14 or they did not meet the inclusion criteria. 15 16 17 18

Two articles 10 11 by the same authors of one of the included articles 19 were excluded, despite having a larger number of patients, because it did not contain the sample specifications that would enable us to calculate the accuracy of MTL-P300 in defining the EZ, and it was not possible to recover these data.

Three studies 7 19 20 were chosen because they met the inclusion criteria and were evaluated according to the JBI scale as containing the data required for a good analysis of internal quality ( Table 1 ).

The included works comprised a total sample of 112 patients. In two studies, 7 20 a consecutive series of patients who had the MTL-P300 registered using depth electrodes was included. In another study, 19 a series of patients who had the MTL-P300 and AMTL-N400 cognitive potentials recorded through stereo-EEG were included.

The gender distribution was 52 women and 60 men; 59 patients had left TLE and 53, right TLE, and the average age was of 28.9 (range: 16–56) years. In the study by Puce et al., 7 there were 33 cases of left TLE and 24 of right TLE in a sample of 32 men and 25 women with a mean age of 27.8 ± 11.5 years. In the study by Meador et al., 20 there were 14 cases of left TLE and 16 of right TLE in a sample of 16 women and 14 men with an average age of 31 (range: 17–56) years. And in the study by Grunwald et al., 19 there were 12 cases of left TLE and 13 of right TLE in a sample of 11 women and 14 men with a mean age of 28 ± 10.5 years. These three studies were performed in the United States, 20 Australia, 7 and Germany. 19 The main characteristics of the articles are described in Table 2 .

**Table 2:** Description of the methods applied in included studies

Author and year	Study design	Population	Intracranial electroencephalography method	Oddball task
Puce et al., 1989 7	Cross-sectional study	57 patients	Bilateral depth electrodes were implanted stereotaxically in the hippocampi via an orthogonal approach	Auditory oddball paradigm
					Meador et al., 1992 20	Cross-sectional study	30 patients	Bilateral depth electrodes placed stereotaxically via a vertex trajectory to traverse the hippocampi	Auditory oddball paradigm
Grunwald et al., 1995 19	Cross-sectional study	25 patients	Bilateral depth electrodes implanted stereotaxically along the longitudinal axis of the hippocampus	Visual oddball paradigm					

In all three studies there was stereotaxic implantation of depth electrodes to reach the hippocampus bilaterally in each patient. The method to obtain cognitive evoked potentials was a standard oddball in the three studies, with the auditory stimulus in two studies 7 20 and the visual stimulus in the other. 19 All studies aimed to analyze the accuracy of the MTL-P300 in detecting the laterality of the seizures in TLE patients. Two studies 19 20 involved patients with unilateral TLE, and the third, 7 patients with different types of drug-resistant epilepsies were included, with an analysis separating the groups and another analysis of the whole sample regarding unilateral TLE.

The sensitivity of the hippocampal evoked potential described by Puce et al. 7 was of 46/57 (81%), and the positive likelihood ratio (LR + ) was of 15.3; the sensitivity found by Meador et al. 20 was of 21/30 (70%) and the LR + , of 2.3; and in the study by Grunwald et al., 19 the sensitivity was of 20/25 (80%), and the LR + , of 4.0. Considering the greater accuracy from the LR+ analysis that takes into account the probability of a positive test being a true positive, we found the method described by Puce et al., 7 then by Grunwald et al., 19 and, finally, the method described in Meador et al. 20 ( Table 3 ). The three studies showed similar values for the negative likelihood ratio (LR-) values, between 0.2 and 0.5, showing little accuracy of a negative test to rule out the diagnosis.

**Table 3:** Description of the outcomes and results of the included studies

Authors	Outcome measures	Results
Puce et al., 1989 7	The ratio of the lowest amplitude of hippocampal MTL-P300 to the largest	Bilaterally absent MTL-P300 only in 7% of the patients; it is not a useful marker to ward off bitemporal epilepsies.
		Sensitivity of abnormal MTL-P300 responses of 81% and specificity of 94,7%; LR+ of 15.3 and LR- of 0.20
Meador et al., 1992 20	Calculation of the sum of the entire power spectrum (0–30 Hz) on the right and left sides during the target stimulus	Lower sensitivity in left TLE than in right TLE. Sensitivity of abnormal MTL-P300 responses of 70% and specificity of 70%; LR+ of 2.3 and LR- of 0.43.
Grunwald et al., 1995 19	Subtracting (the amplitude of the MTL-P300 on the left minus the amplitude on the right)	The sensitivity of the MTL-P300 was not significant to diagnose EZ laterality in patients without hippocampal sclerosis.
		Sensitivity of abnormal MTL-P300 responses of 80% and specificity of 80%; LR+ of 4.0 and LR- of 0.25.

Abbreviations: EZ, epileptogenic zone; LR-, negative likelihood ratio; LR + , positive likelihood ratio; MTL-P300, medial temporal lobe P300; TLE, temporal lobe epilepsy.

In the three articles, 7 19 20 the amplitude of the MTL-P300 was calculated in relation to the baseline from 200 ms to 500 ms before the stimulus. The measure of amplitude reduction to detect the MTL-P300 side as abnormal was different in these three studies. In one of the articles, 7 the amplitude of the MTL-P300 drop side considered abnormal was predicted when there was a ratio between the side with reduced amplitude and the opposite side lower than 0.5; therefore, the side with the smallest amplitude was considered ipsilateral to the EZ. Then, this classification was correlated with the findings of the seizure recordings. There was no analysis of the sensitivity of the MTL-P300 in detecting seizures on the left and on the right sides separately.

In the study by Meador et al., 20 the spectral power of the MTL-P300 frequencies was analyzed in both hippocampi: the side with reduced spectral power was considered ipsilateral to the EZ, and these findings were compared with the lateralization of the EZ obtained from the seizure recordings. The sensitivity of the results for patients with seizures on the right and on the left sides was obtained using analysis of variance (ANOVA), with a proportion of correct answers of 88% for patients with seizures on the right and of 50% for those with seizures on the left.

Finally, in the study by Grunwald et al., 19 the laterality of the EZ was determined by subtracting the left and right amplitudes of the MTL-P300, with a sensitivity of 83% for seizures on the left side, and of 77% for seizures on the right side; therefore there is no important difference between the sides in which the seizures occur.

The three studies 7 19 20 classified the neuropathological findings regarding the presence of hippocampal gliosis and other findings, and none of them had findings on magnetic resonance imaging (MRI). Two of these studies 7 19 evaluated the results of the MTL-P300 in relation to patients who had histopathological findings of hippocampal gliosis versus the others. In Puce et al., 7 the sensitivity of the abnormal MTL-P300 response due to the presence of hippocampal sclerosis was of 100%. Among the 22 hippocampi investigated, 13 had hippocampal sclerosis, and all of them had an absent MTL-P300. In total, the 9 hippocampi presented normal results on the anatomopathological study, 5 had absent MTL-P300, and 4 had normal MTL-P300, but the authors did not describe in how many of the 5 cases of absent MTL-P300 there was agreement between the laterality of the EZ and the MTL- P300, and it is not possible to calculate the sensitivity of the correct MTL-P300 in this small sample without hippocampal sclerosis. Using the Pearson Chi-squared test ( *p* < 0.05), Grunwald et al. 19 found an association between the reduction in amplitude of the MTL-P300 in the group with hippocampal sclerosis. In the group without hippocampal sclerosis, among 8 hippocampi in the EZ, only 4 showed a reduction in the amplitude of MTL-P300, and no statistically significant association was found. In Meador et al., 20 the percentage of cases with gliosis was described among patients with TLE on the left and right sides, with no analysis of the correctness in terms of the groups with gliosis versus the others.

## DISCUSSION

The results of the present systematic review show that the abnormal hippocampal evoked potential MTL-P300 has high LR + , which is compatible with a high accuracy in detecting the laterality of the EZ in unilateral TLE, 7 19 and a LR- compatible with a low accuracy to rule out this diagnosis when the test is normal. Two studies 7 19 found a high specificity, of 94.7% and 80% respectively, demonstrating that the MTL-P300 is an additional tool to determine the lateralization of the EZ. Future studies with a larger number of patients could clarify whether the MTL-P300 can be used as a marker of EZ in patients without hippocampal sclerosis.

Puce et al. 7 suggest that this potential has low sensitivity in detecting the involvement of the contralateral hippocampus, evidenced by a small rate of only 7% of abnormal MTL-P300 bilaterally. Only Meador et al. 20 suggested a lower rate of sensitivity of the method in the left TLE, a finding not reported in the other studies. A high rate of abnormal MTL-P300 was found in hippocampi with hippocampal sclerosis in two studies, 19 with no significance between the number of abnormal MTL-P300 and EZ in the group without hippocampal sclerosis. It is worth mentioning that the sample of patients without hippocampal sclerosis was small.

In another article, Grunwald et al. 11 analyzed 84 patients, 29 of whom without hippocampal sclerosis, and found reduced amplitudes of the MTL-P300 on the side of the EZ only in the group of patients with hippocampal sclerosis. We believe that an analysis of a larger sample of patients without hippocampal sclerosis is necessary to better represent the different severity stages of TLE; then physicians will be able to analyze the usefulness of this method in detecting hippocampal dysfunction without histological injury.

The three articles 7 19 20 included clearly described the inclusion criteria and objective of the studies, in addition to using an appropriate methodology to investigate and quantify the results, showing a good accuracy of the methods in detecting the epileptogenic zone in unilateral TLE. The study by Puce et al. 7 showed an LR + higher than 10, which corresponds to an optimal accuracy, meaning that abnormal results have a high probability of corresponding to true positives; the study by Grunwald et al. 19 showed moderate accuracy for positive tests, and the method used by Meador et al., 20 little accuracy for positive tests. All three had LR- compatible with a low accuracy for the negative test to rule out the diagnosis. The low sensitivity and LR- show us that the presence of the MTL-P300 does not exclude the epileptogenicity of the hippocampus. Tests with LR- close to 0 (< 0.1) are good diagnostic tests. In other words, when the test result is negative, the lower the probability of illness.

The studies that met the selection criteria to answer our main question, whether the MTL-P300 is a good marker of the EZ, do not provide information regarding the correlation between verbal performance and the evoked potentials of the hippocampus. However, one of the excluded articles, also by Grunwald et al. 10 –the reasons were mentioned in the “Methods” section –, analyzed 40 patients who underwent left hippocampal resection.

It described a decrease in the amplitude of the MTL-P300 responses on the side of the EZ, but there was no correlation regarding the responses of the MTL-P300 on the right and on the left sides with the performance in verbal memory in the postoperative period. Although this study did not show a significant correlation between the MTL-P300 and performance in verbal memory cognitive tests, we observed that there was no analysis between these cognitive potentials and performance in tests of non-verbal memory. Therefore, in the present review, the study by Grunwald et al. 10 was the only that correlated the MTL-P300 with postoperative verbal memory performance. The functional preservation of the hippocampus evidenced through a normal MTL-P300 in a specialized hemisphere for language could be linked to an increased risk of dysfunction in verbal memory in case of surgery of this temporal lobe. Likewise, the abnormal MTL-P300 response in the right hippocampus contralateral to the epileptogenic zone in the left hippocampus may indicate an increased risk of dysfunction in postoperative verbal memory due to the lack of functional reserve.

Still in this study, 10 the temporal lobes were analyzed using the AMTL-N400 potential, and a significant correlation was found between the amplitudes of the responses of the right rhinal cortex and the performance of the delayed verbal recall test. These findings indicate that the greater the functional integrity of the right rhinal cortex, the more likely it will be able to compensate for the loss of functionality with the resection of the mesial structures of the left temporal lobe. Although no correlation was found in this study 10 , their findings suggest that the greater the postoperative AMTL-N400 response in the left rhinal cortex, the greater the decrease in performance in verbal memory in the postoperative period.

A similar correlation between MTL-P300 amplitude and performance in memory tests in the postoperative period would be necessary to answer our secondary question, but this was not found. Based on the study by Puce et al., 7 we can predict that the MTL-P300 has low sensitivity in detecting the involvement of the contralateral hippocampus because it is not a good marker of bilateral TLE. Therefore, the MTL-P300 does not aid in defining surgical prognosis in terms of seizure control.

We can conclude that, when the MTL-P300 is abnormal, there is high specificity in detecting the EZ in TLE. The MTL-P300 responses do not contribute to a prognosis of the postoperative control of seizures, as it does not detect cases of bitemporal epilepsy. Neither does it seem to be a good indicator of verbal memory performance. Our review showed a limitation in answering our second question. New studies could assess whether the MTL-P300 is a good marker in verbal memory before and after surgery. Due to the importance of the functional analysis of the hippocampus, further studies are needed to clarify the correlation involving cortical dysfunction in the hemisphere specialized in language and in the contralateral hemisphere and the findings obtained through this marker of hippocampal functionality.
